# Genetic, structural, and functional characterization of allomelanin from black yeast *Exophiala viscosa*, a chassis for fungal melanin production

**DOI:** 10.1007/s00253-025-13597-w

**Published:** 2025-10-08

**Authors:** Tiffany M. Hennessa, Lauren M. Irie, Hong Dong, Eric S. VanArsdale, Evan R. Glaser, Erin C. Carr, Steven D. Harris, Nathan C. Gianneschi, Zheng Wang

**Affiliations:** 1 Center for Bio/Molecular Science and Engineering, Naval Research Laboratory, Washington, DC 20375 USA; 2 Department of Materials Science and Engineering, Northwestern University, Evanston, IL 60208 USA; 3 International Institute for Nanotechnology, Chemistry of Life Processes Institute, Simpson Querrey Institute, Northwestern University, Evanston, IL 60208 USA; 4 Biological and Biotechnological Sciences Division, DEVCOM, Army Research Laboratory, Adelphi, MD 20783 USA; 5 Electronics Science and Technology Division, Naval Research Laboratory, Washington, DC 20375 USA; 6 School of Biological Sciences, University of Nebraska-Lincoln, Lincoln, NE 68588 USA; 7 Department of Plant Pathology, Entomology and Microbiology, Iowa State University, Ames, IA 50011 USA; 8 Department of Chemistry, Northwestern University, Evanston, IL 60208 USA; 9 Department of Biomedical Engineering, Northwestern University, Evanston, IL 60208 USA

**Keywords:** *Exophiala*, Melanized fungus, DHN-melanin, Allomelanin, Biomanufacturing, Radiation resistance

## Abstract

**Abstract:**

Melanized fungi are known for their remarkable resilience to environmental stress, largely attributed to the protective properties of melanin. In this study, we establish the black yeast *Exophiala viscosa* as a non-pathogenic, genetically tractable model for the scalable production and functional analysis of DHN-melanin (allomelanin). Cultivation in flasks and bioreactors yielded up to 8.6 g/L of melanin, with the majority tightly incorporated into the cell wall as “melanin ghosts”. Chemical analyses including FTIR, XPS, ssNMR, and EPR confirmed the identity of the pigment as allomelanin and revealed a structural association with chitin. Gene deletions of Pks1, Arp2, and Abr2 validated the DHN-melanin biosynthetic pathway and enabled the generation of pigment-deficient mutants. Functional assays demonstrated that melanin contributes significantly to UV and cold tolerance, while offering limited protection against γ-radiation, suggesting that other pigments,such as carotenoids, may also play a protective role. The unique redox properties, structural integrity, and scalability of melanin production in *E. viscosa* highlight its potential for bio-derived materials used in radiation shielding, environmental remediation, and thermal regulation. This work establishes *E. viscosa* as a promising chassis for melanin biomanufacturing and a valuable model for studying fungal melanins in the context of materials science and environmental resilience.

**Key points:**

• *Cultivation of E. viscosa in rich medium yielded up to 8.6 g/L of melanin.*

• *Chemical and genetic analyses identified the pigment as allomelanin.*

• *Melanin enhanced the tolerance of fungal cells to UV radiation and low temperatures.*

**Supplementary Information:**

The online version contains supplementary material available at 10.1007/s00253-025-13597-w.

## Introduction

Melanized fungi are a diverse group of microorganisms ubiquitously distributed across a wide range of habitats and are characterized by the production of melanin, a heterogeneous biopolymer that provides various protective benefits (Gostincar et al. 2012; Mattoon et al. 2021). The presence of melanin in fungal cell walls has been associated with resistance to environmental stressors, such as UV radiation, ionizing radiation, extreme temperatures, and toxic heavy metals (Cordero et al. 2018; Dadachova and Casadevall 2008; Gadd and Derome 1988; Gostincar et al. 2012; Kejzar et al. 2013; Onofri et al. 2008; Selbmann et al. 2015). Among melanized fungi, species within the genus *Exophiala* are of particular interest due to their remarkable resilience and unique metabolic capabilities. The genus *Exophiala*, belonging to the order Chaetothyriales, encompasses a group of black yeasts and filamentous fungi known for their adaptability to diverse ecological niches, with numerous species implicated in cutaneous and superficial infections in humans and animals (Zeng et al. 2007). In addition, many *Exophiala* species are oligotrophic. They thrive in nutrient-poor environments, and exhibit remarkable versatility, colonizing substrates such as hydrocarbon-rich soils, decaying organic matter, and even extreme habitats like polluted wastewater (De Hoog et al. 2006; Ide-Pérez et al. 2020; Ide-Perez et al. 2020; Li et al. 2020; Woertz et al. 2001). In nature, *Exophiala* species play key ecological roles in nutrient cycling, bioremediation of toxic compounds, and symbiotic interactions with plants and other organisms (Li et al. 2011; Seyedmousavi et al. 2011). Their ability to degrade hydrocarbons and sequester heavy metals further highlight their importance in environmental systems. Prominent species within this genus, such as *Exophiala dermatitidis* and *Exophiala lecanii-corni*, have been extensively studied for their resistance to environmental stressors (Chen et al. 2014; Romsdahl et al. 2021). *Exophiala dermatitidis* is particularly notable for its high tolerance to ionizing radiation, with a D10 (dose reducing survival to 10%) exceeding 3000 Gy, making it one of the most radiation-resistant eukaryotes (Schultzhaus et al. 2020). Similarly, *E. lecanii-corni* demonstrates significant UV and oxidative stress resistance (Romsdahl et al. 2021), underscoring the protective role of melanin in these species. There is increasing evidence that this stress tolerance is closely associated with the physicochemical properties found in melanin. Genomic and genetic analyses in these studies also revealed the production of DHN-melanin, also known as allomelanin, which is synthesized via the polyketide pathway.

Allomelanin is a unique type of melanin distinct from eumelanin and pheomelanin found in animals (Lino and Manini 2022). It is derived from 1,8-dihydroxynaphthalene (DHN) through a series of enzymatic reactions catalyzed by polyketide synthases (PKS) and accessory enzymes such as scytalone dehydratase and laccase (Heinekamp et al. 2012; Tsai et al. 1999). This pathway is widespread among ascomycetous fungi and is critical for producing melanin with distinctive physicochemical properties. Allomelanin's structure, characterized by its aromatic polymeric network, imparts unique features such as high UV absorption, metal-binding capacity, and exceptional stability under extreme conditions (Lino and Manini 2022; McCallum et al. 2021). These properties make allomelanin highly attractive for applications in biomaterials, radiation shielding, and environmental remediation.

Despite its promising attributes, the scalable production of allomelanin remains challenging. This is primarily due to limited natural sources and low yields in traditional systems. Black yeasts, particularly those within the *Exophiala* genus, are ideal candidates for allomelanin production because of their natural ability to synthesize high levels of melanin and their robust growth under laboratory conditions. However, while *E. dermatitidis* and *E. lecanii-corni* have been well studied, their features, such as pathogenicity and filamentous development, limit their suitability as industrial chassis (Chen et al. 2014; Romsdahl et al. 2021). Within this context, *Exophiala viscosa* has emerged as a promising model organism owing to its non-virulent nature, ease of cultivation, and predominantly yeast-like morphology (Carr et al. 2023). Previous reports suggest that it can produce significant quantities of melanin and even actively secrete melanin, making it an excellent candidate for investigating allomelanin biosynthesis and function. Although genomic and genetic studies of *Exophiala* species have referred to their synthesized melanin as DHN-melanin or allomelanin, its chemical composition and structure remain inadequately defined and undercharacterized.

The motivation of this study is to: (1) utilize *E. viscosa* as a model to produce and characterize DHN-melanin; (2) investigate the role of DHN-melanin in protecting cells against environmental stressors such as radiation and extreme temperatures; and (3) demonstrate the potential of *E. viscosa* as a platform for genetic engineering to enable the manipulation of melanin biosynthetic pathways to enhance production or tailor melanin's properties for specific applications. This study not only seeks to deepen our understanding of fungal melanins but also aims to leverage the unique properties of allomelanin for novel applications in materials science and environmental sustainability.

## Materials and methods

### Cultivation of *E. viscosa*

*Exophiala viscosa* JF 03-3F CBS 148801 strain, unless otherwise noted, was cultured at 25 °C in liquid or solid yeast peptone dextrose (YPD) (10 g/L yeast extract, 20 g/L peptone, 20 g/L glucose), with liquid cultures shaking at 200 RPM. For melanin production in bioreactor, freshly cultured *E. viscosa* cells were inoculated at 10^4^ cells/mL into a 4 × 100 mL YPD in a DASGIP bioreactor (Eppendorf). Bioreactor process parameters were set for a temperature of 25 °C, pH set point of 7.0, and a dissolved oxygen (DO%) setpoint of 30%, and the experiment was run for 7 days. DO%, pH, and foam were automatically controlled as described in our previous publication (Smith et al. 2023).

### Isolation of fungal melanin

Isolation and purification of fungal melanin and melanin ghosts were performed using an acid/base cycling protocol as described in our previous studies (Romsdahl et al. 2021; Smith et al. 2023). Briefly, fungal cultures were centrifuged at 6,000 × g for 10 min, the melanin-containing supernatant was transferred to a separate flask, and fungal cell pellets were kept for the isolation of melanin ghosts. Melanin in the supernatant was precipitated by acidifying the solution with 6 M HCl until the pH was reduced to about 2. The precipitated melanin was then collected by centrifugation at 10,000 × g for 10 min. The acidified supernatant was discarded and the melanin pellet was resuspended in 1 M NaOH and incubated in a water bath at 80 °C for 1 h. After cooling to room temperature, melanin was re-precipitated by adding 6 M HCl to adjust the pH below 2, followed by a 30-min incubation at room temperature. This acid/base cycling procedure was repeated for a minimum of three cycles. Following the final cycle, the melanin precipitate was washed three times with water, then dried in a 65 °C oven for 24–48 h, yielding dry melanin granules. Melanin ghosts were prepared by subjecting fungal cell pellets to the same acid/base cycling procedure to remove the majority of cellular components, followed by drying in a 65 °C oven for 24–48 h. The resulting cell wall scaffolds, largely composed of melanin, are referred to as melanin ghosts. Both dried melanin granules and ghosts were weighed using a precision scale.

A second round of extraction was performed on the melanin granules to remove as much of the chitin contaminants as possible. The acid–base extraction procedure followed a previously published protocol(Singla et al. 2021) with some modifications. Briefly, 100 mg of pristine fungal melanin granules were dissolved in 4 mL of 1 M NaOH and left to sit for 30 min to allow for solubilization of melanin in the basic solution. Next, the solution was autoclaved (120 °C, 45 min). The solution was then centrifuged and the supernatant was collected. Concentrated HCl was added dropwise to the supernatant until the solution reached a pH of 1, which will allow for the melanin to precipitate out of the supernatant. The precipitated melanin was then collected via centrifugation. Lastly, the precipitate was acid refluxed at 120 °C for 20–24 h and recollected after centrifugation with three water washes, and one ethanol wash.

### Scanning electron microscopy imaging

Fungal cells and melanin ghosts were fixed in 4% paraformaldehyde aqueous solution for 2 h. Following fixation, the samples (including melanin, melanin ghosts, and fungal cells) were stained with 1% phosphotungstic acid aqueous solution (Electron Microscopy Sciences) for 2 h, then spun down, freeze-dried, and sputter coated with 5 nm gold. Finally, samples were imaged using a FEI Quanta 200 F field emission scanning electron microscope (FESEM) at an accelerating voltage of 5 kV.

### Genetic manipulation

*Exophiala viscosa* mutant strains, *Evpks1Δ*, *EvAbr2Δ*, and *EvArp2Δ*, were generated using established gene deletion techniques with some modifications in black yeast described by Romsdahl (Romsdahl et al. 2021). Each gene deletion was accomplished via homologous recombination, in which the target gene was replaced by a hygromycin resistance cassette (hph) using the double-joint PCR method. Construct assembly was performed using Phusion Flash High-Fidelity PCR Master Mix (Thermo Fisher Scientific, Waltham, MA) following the manufacturer’s guidelines. Upstream flanking regions were amplified using primers F1 and R3 and downstream flanking regions using primers F4 and R6. The final deletion cassette, consisting of the hph resistance marker flanked by homologous arms, was assembled using primers F2 and R5. Sequences of PCR primers are listed in Table S1.

To generate electrocompetent cells in *E. viscosa*, a unique transformation protocol combining protoplast generation with electroporation was performed (Zhao et al. 2019). Briefly, *E. viscosa* cells (~ 10^8^ cells/mL) were inoculated into 50 mL of MEA Liquid media and incubated at 25 °C for 24 h, shaking at 200 rpm. Yeast cells were harvested by filtration through sterilized Miracloth sheets and subjected to enzymatic digestion in 35 mL of VinoTaste protoplast solution and incubated at 35 °C for 1-3 h while shaking. Undigested material was removed through filtration and protoplast were washed with 0.6 M KCl/50 mM CaCl₂ and then with ice-cold 1 M sorbitol. Washes were performed at 4 °C and protoplast concentration was estimated using a hemocytometer to ensure a final concentration of ≥ 10⁸ protoplasts/mL.

For transformation, 50-100 μL of protoplast suspension was mixed with 4-5 μg of linearized DNA (volume ≤ 10 μL) and transferred to a chilled 0.2 cm electroporation cuvette (Bio-Rad, Hercules, CA). Following a 30-min incubation on ice, cells were electroporated using a Bio-Rad Gene Pulser (Bio-Rad, Hercules, CA) under the fungal setting and pulsed twice. Immediately after pulsing, 800 μL of pre-warmed YPD recovery medium was added to the cuvette. The mixture was transferred to a 15 mL tube and incubated at a shaking incubator at 25 °C for 2–3 h. Cells were then spread onto MEA agar plates supplemented with 50 μg/mL of hygromycin B and incubated at 25 °C. Transformants appeared after 5–10 days and were wrapped in foil to prevent desiccation. Successful transformants were screened by PCR to confirm gene deletion (Fig. S1).

### Cell survival and sensitivity assays

For radiation resistance and stress sensitivity assays, cells of *E. viscosa* wild-type and *Evpks1* mutant were cultured in 2.5 mL YPD in 15 mL culture tubes for 6 days. After the growth, cells were re-suspended to a concentration of 10^8^ cells/mL in cold water and placed on ice. For UV-C exposure, cells were plated on YPD solid media and exposed in triplicate to either 0, 25, 50, or 75 mJ/cm^2^ of UV-C Light within a biosafety cabinet with a dose rate of 0.273 mJ cm^−2^ s^−1^. For γ-radiation exposure, 100 μL of cells were aliquoted to PCR tubes, which were exposed to either 0, 500, 1000, or 1500 Gy of γ-radiation by a Cobalt-60 source with a dose rate of approximately 22.7 Gy/min. For each sample and dose, three biological replicates were exposed to the radiation source and each biological replicate was plated onto two YPD plates following the irradiation. The relative survival was evaluated by counting colony forming units (CFUs). The standard deviations were generated with Microsoft Excel. Temperature sensitivity was evaluated by spotting 2 μL droplets of cells of serially diluted cell suspensions (10⁸ cells/mL, 10⁰ to 10⁻⁶) onto solid YPD plates. Three biological replicates were incubated at 4 °C, 25 °C, and 30 °C, respectively. Colony images were captured after 26 days of incubation at 4 °C and after 7 days at 25 °C and 30 °C.

### Synthesis of synthetic allomelanin (p-1,8-DHN)

Synthetic allomelanin (p-1,8-DHN) was synthesized based on a previously published protocol (Zhou et al. 2019). 1,8-dihydroxynapthalene (1,8-DHN) (95 + %) was purchased from Matrix Scientific. Briefly, 150 mg of 1,8-DHN was added to 7.5 mL of acetonitrile (ACN) and stirred for a few minutes to dissolve before adding 142.5 mL of ultrapure water to the mixture. The reaction mixture was injected with a 1 mL solution containing NaIO_4_ (0.47 mmol, 100.15 mg) and stirred for 20 h before being centrifuged and washed with ultrapure water (11,000 rpm, 10 min) three times.

### Chemical analysis of fungal melanin

X-ray photoelectron spectroscopy (XPS): XPS spectra were acquired with a Thermo Scientific Nexsa G2 system. Spectra deconvolution was performed using Advantage software (version 5.975) employing Smart background subtraction and peak fitting by means of the Simplex algorithm.

Fourier Transform Infrared Spectroscopy (FTIR): FTIR spectra were collected with a Thermo Scientific Nicolet iS50 spectrometer.

Solid state nuclear magnetic resonance (ssNMR): Samples (40–50 mg) were packed into a 4.0 mm magic-angle spinning (MAS) rotor from Bruker. ^13^C{^1^H} multiple cross-polarization magic angle spinning solid-state nuclear magnetic resonance (^13^C multi-CPMAS ssNMR) spectra were recorded on a Bruker Advance III 400 MHz spectrometer equipped with a Bruker 4 mm HX MAS probe. The protocol for acquisition was based on a previous publication (Johnson and Schmidt-Rohr 2014). The π/2-pulse lengths were 2.1 μs for 1H and 3.65 μs for 13 C and the 1H decoupling field strength was 119 kHz during signal detection. The MAS rotation frequency was set to 14 kHz, with a relaxation delay of 1 s (T_1H_, the spin–lattice relaxation time of proton, is about 0.5 s) incorporated within the multiple CP ramp, each having a contact time of 0.5 ms. ^13^C{^1^H} multiple CPMAS (multi-CPMAS) spectra were recorded with 1024 transients. The recycle delay was set for 1 s. The proton spin relaxation in the rotating frame (T_1ρH_) was found around 8000 μs.

### Electron paramagnetic resonance (EPR)

The EPR spectra were acquired at 300 K using a commercial (E-300) Bruker Biospin spectrometer operating at a frequency of 9.51 GHz. Several milligrams of fungal melanin granules, melanin ghosts, and eumelanin synthesized from bacteria (Smith et al. 2023) were put in 4-mm OD low-loss quartz tubes with the tubes inserted in the middle of the cylindrical microwave cavity. The number of spins/mg was determined by double integration of the EPR signals and comparison with a well-calibrated P-doped Si standard.

### Reverse electrochemical mediated probing

For electrochemical testing, melanin-chitosan films were prepared on gold electrodes as described previously with slight modifications (Kang et al. 2018). Briefly, 0.5% chitosan solution (pH of 5.5) was mixed with melanin powder to a concentration of 1 g/L. 20 μL of the solution was cast on to the electrode and then vacuum dried for 15 min. The film was then neutralized in phosphate buffer (0.1 M, pH 7.5) for 10 min. Empty chitosan films were used as controls. The films were next probed using 100 μM phenazine-1-carboxylic acid (PCA) and 50 μM ferrocene dimethanol (FC) as our reductive and oxidative mediators, respectively. Cyclic voltammetry sweeps were done with modified gold electrodes in the previously described set-up (Kang et al. 2018; VanArsdale et al. 2025) between −0.65 to + 0.5 V vs Ag/AgCl at a scan rate of 2 mV/s. All measurements were performed using a CH Instruments 720E potentiostat (CH Instruments).

## Results

### Extraction of melanin from *E. viscosa* culture

The previous study demonstrated that yeast peptone dextrose (YPD) medium stimulated the release of melanin from *E. viscosa*, with 2% peptone identified as the critical factor for inducing melanin excretion (Carr et al. 2023). To optimize melanin production, *E. viscosa* was cultivated in 100 mL of YPD medium at 25 °C for 7 days in both flasks and bioreactors. Melanin was subsequently extracted from the culture supernatants and cell pellets, resulting in two distinct products: melanin granules and melanin ghosts, respectively.

**Fig. 1:**
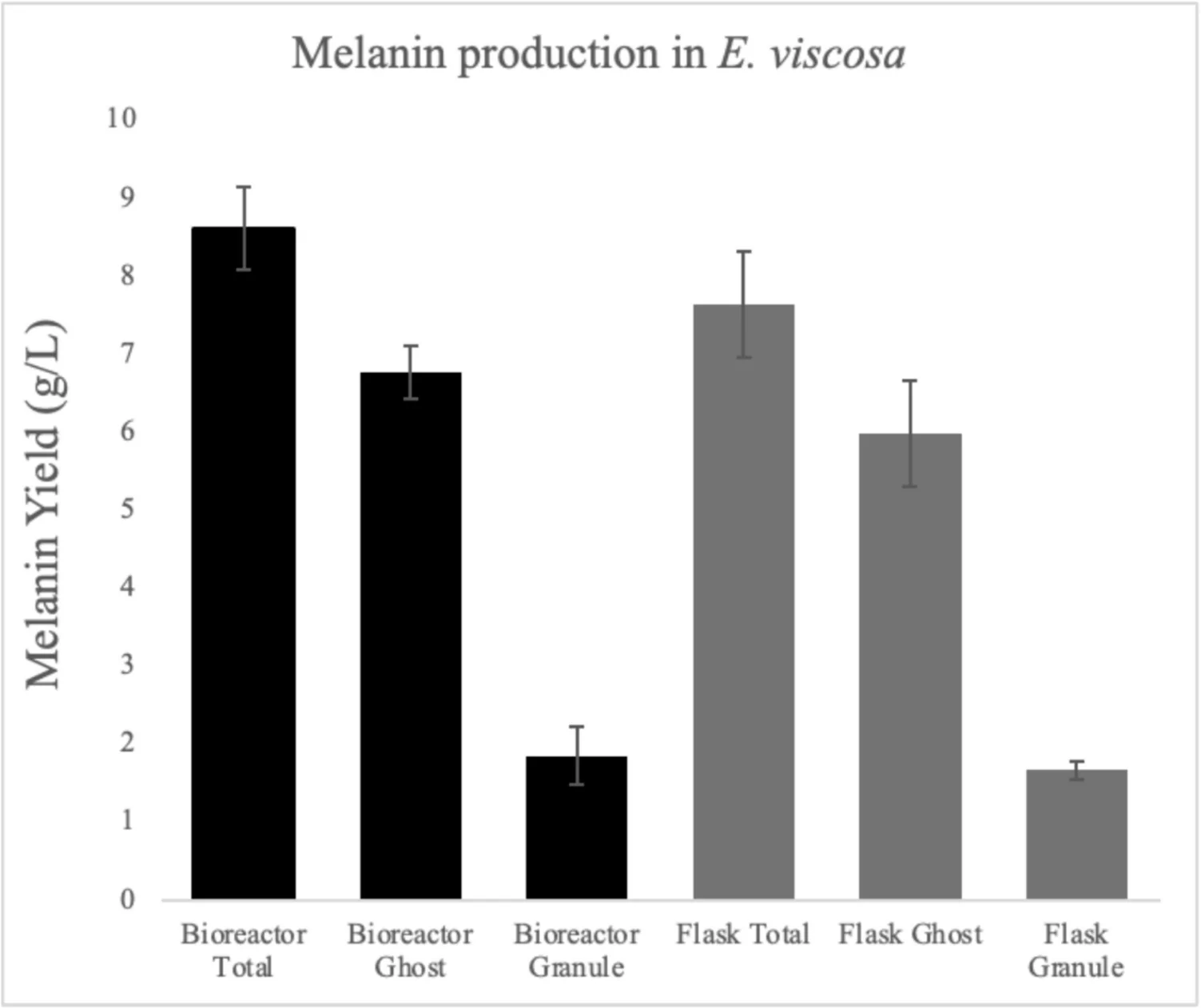
Melanin production from *E. viscosa* in bioreactor and flask. Melanin yields (g/L) were generated from four biological replicates under each growth condition

Melanin yields were approximately 8.62 ± 0.54 g/L from bioreactor cultures and 7.64 ± 0.68 g/L from flask cultures (Fig. 1). Although the total melanin yield was slightly higher in the bioreactor, a larger proportion of melanin was consistently recovered as melanin ghosts, 6.8 ± 0.3 g/L in bioreactors and 6.0 ± 0.7 g/L in flasks, suggesting that the majority of melanin is tightly incorporated within the fungal cell wall. In contrast, melanin granules precipitated from secreted melanin in the culture supernatant were recovered at lower concentrations: 1.9 ± 0.4 g/L in bioreactors and 1.7 ± 0.1 g/L in flasks.

**Fig. 2:**
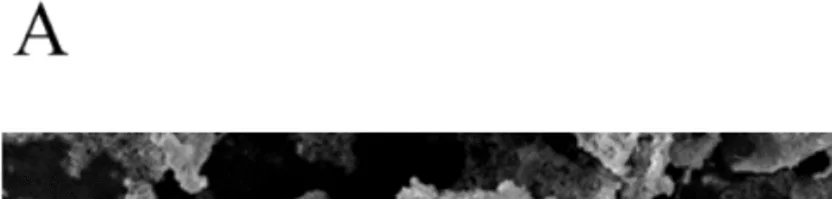
FE-STEM images of fungal melanin granules (**A**) and melanin ghosts (**B**)

Melanin granules were characterized using field emission scanning transmission electron microscopy (FE-STEM), which revealed irregularly shaped, solid aggregates with porous and textured surfaces (Fig. 2A). These structural features are comparable to melanin isolated from the black yeast *E. lecanii-corni*, the black knot fungus *Dibotryon morbosum*, and the mushroom *Auricularia auricula* (Prados-Rosales et al. 2015; Romsdahl et al. 2021; Singla et al. 2021). Fungal melanin is typically assembled within the cell wall, where it plays a critical role in maintaining structural integrity. Melanin associated with the fungal cell wall, often referred to as “melanin ghosts,” is traditionally isolated using harsh chemical treatments, such as 4 M guanidinium isothiocyanate followed by 6 M HCl at 100 °C (Kim et al. 2017; Romsdahl et al. 2021; Wang et al. 1996). In this study, however, melanin ghosts were generated using an acid/base cycling protocol. Scanning electron microscopy (SEM) revealed hollow ghost structures with open budding sites, while the overall architecture of the cell wall layers remained intact (Fig. 2B).

### Chemical characterization of fungal melanin

**Fig. 3:**
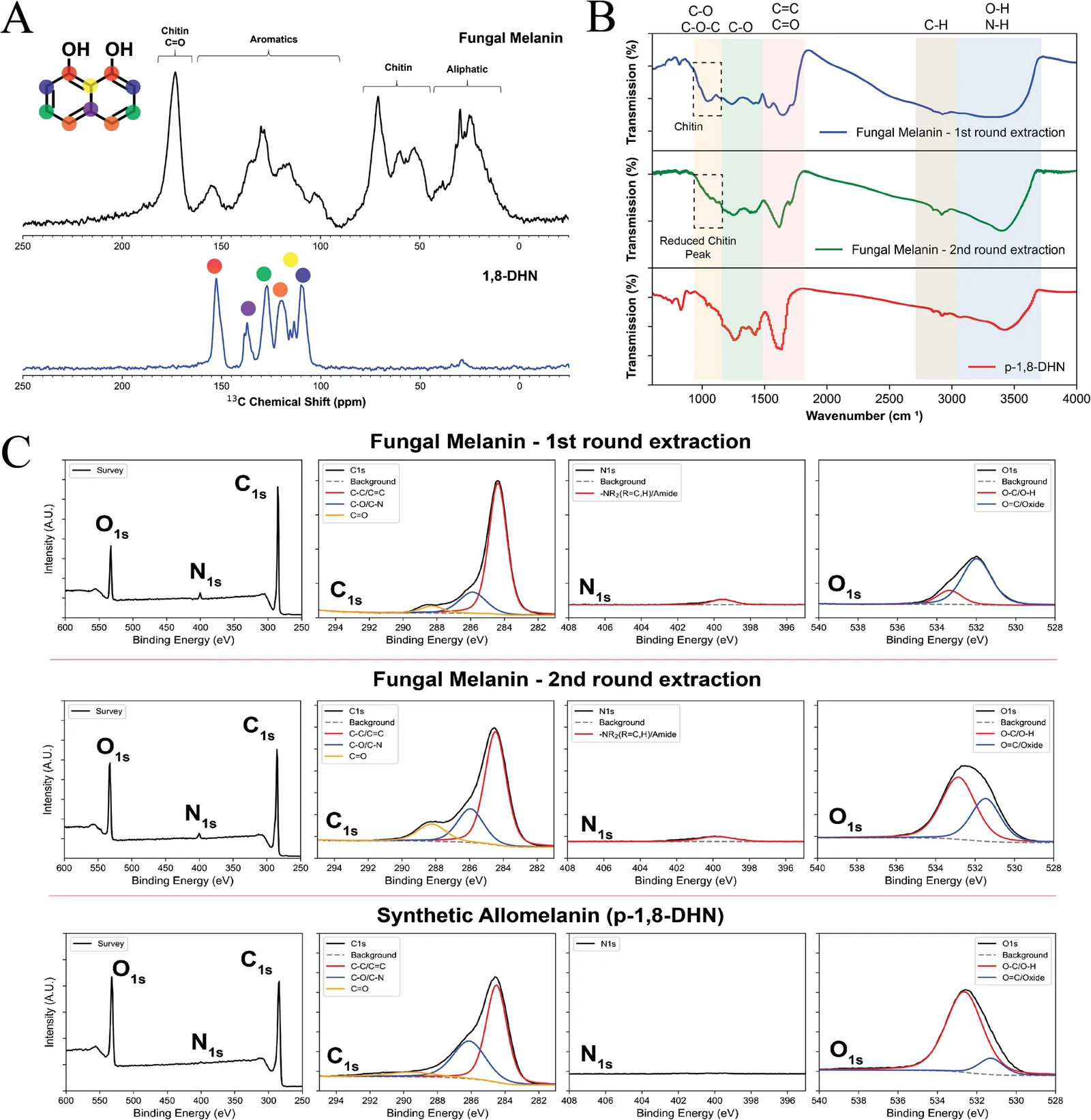
Chemical characterization of fungal melanin. (**A**) ^13^C multi-CPMAS ssNMR spectra of fungal melanin (top) and 1,8-DHN monomer (bottom); (**B**) FTIR spectra of fungal melanin after 1 st and 2nd round of extraction compared to synthetic p-1,8-DHN allomelanin; (**C**) XPS spectra of fungal melanin after 1 st and 2nd round of extraction compared to synthetic p-1,8-DHN allomelanin

A number of spectroscopic techniques were employed to characterize chemical properties of the fungal melanin. To investigate the carbon chemical environment of the isolated fungal melanin, ^13^C multiple cross-polarization magic angle spinning solid-state nuclear magnetic resonance (^13^C multi-CPMAS ssNMR) spectra were acquired to provide a quantitative carbon ssNMR to compare against the 1,8-DHN monomer (Fig. 3A). ^13^C multi-CPMAS ssNMR spectra confirm the presence of chitin as well as aliphatic species present in the fungal melanin granules. The aromatic region exhibits the melanin’s carbon environment with peaks that correspond very well to the 1,8-DHN monomer. These analyses suggest that the melanin extracted from *E. viscosa* cultures is DHN-melanin, also known as allomelanin, but the material is strongly bound to chitin and aliphatic molecules, consistent with findings reported for black knot fungus (Singla et al. 2021).

In an attempt to remove as much of the chitin as possible and further elucidate the chemical identity of the fungal melanin, a second round of extraction involving the use of an autoclave in addition to the acid–base cycling was performed on the fungal melanin sample. The autoclave applies high pressure and temperature to the sample to more effectively dissemble the fungal melanin ghosts so that biomoleucles like chitin and other contaminants can be removed during acid–base extraction. Fourier-transform infrared spectroscopy (FTIR) was used to characterize and compare the functional groups present in the isolated fungal melanin after the 1 st and 2nd extractions. The FTIR spectra of the fungal melanin was also compared against a common synthetic analogue of allomelanin, poly-1,8-DHN (p-1,8-DHN), which is derived from the oxidative homopolymerization of the 1,8-DHN monomer (Zhou et al. 2019). It is evident from comparing the spectra that the two materials exhibit many shared characteristic melanin functional groups, including hydroxyls (–OH), quinoid structures (C = O), and aromatic peaks (C = C) (Fig. 3B). Some distinct differences include a broad peak between 800–1100 cm^−1^ that is present in the fungal melanin after the 1 st extraction method is applied, which have been previously attributed to the C–O (1034, 1069, and 1110 cm^–1^) and C–O–C stretch (1155 cm^–1^) modes of chitin (Ahmad et al. 2022). The presence of chitin supports our observations in the ^13^C multi-CPMAS ssNMR spectra. In contrast, application of the 2nd round of extraction reduces those chitin peaks and the resulting FTIR peaks look much more similar to the p-1,8-DHN synthetic allomelanin sample (Fig. 3B).

Finally, to further confirm that these fungal melanins are indeed nitrogen-free DHN-melanins, x-ray photoelectron spectroscopy (XPS) analysis was utilized to characterize the elemental composition of the fungal melanin. The XPS of the fungal melanin after the 1 st and 2nd extractions were also characterized (Fig. 3C, Table 1). The XPS data of the fungal melanin after the 1 st round of extraction shows an elemental composition of primarily carbon, nitrogen, and oxygen at 82%, 3%, and 15%, respectively (Fig. 3C). The presence of nitrogen indicates that chitin, polysaccharides, and/or proteins are conjugated to the melanin in agreement with the ssNMR data. In contrast, the 1,8-DHN synthetic allomelanin shows an elemental composition of primarily carbon and oxygen at 77% and 23%, respectively (Fig. 3C). The addition of a 2nd round of extraction using the autoclave confirms the removal of much of the chitin as the atomic percent of carbon decreases form 82% to 75% and the nitrogen decreases from 3 to 2%, which is also reflected in the FTIR measurements (Fig. 3C, Table 1). Furthermore, the fungal melanin after the 1 st round of extraction showed a higher proportion of O = C/oxide bonds over O–C/O–H in contrast to the p-1,8-DHN, which can be attributed to chitin and lipid molecules as well as metal oxide/carbonate contaminants which can appear around 529–532 eV. Application of the 2nd round of extraction decreased this proportion of O = C/oxide bonds over O–C/O–H in the fungal melanin sample to a ratio more similar to that of p-1,8-DHN indicating enhanced contaminant removal. The low nitrogen population of 2% after the 2nd round of extraction shows that this melanin is indeed nitrogen-free with an XPS elemental composition much more similar to the synthetic p-1,8-DHN allomelanin. For reference, L-DOPA-based natural melanins like sepia melanin tend to have nitrogen atomic percents of around 10% (Singla et al. 2021). We acknowledge, however, that fungal melanins can spontaneously react with phenolic monomers from its environment (Baker et al. 2022; Bell and Wheeler 1986) Thus, it is still possible that these fungal melanins could exhibit nitrogen-containing monomers like L-DOPA or other nitrogen-containg biopolymers covalently bound within its internal structure.

**Table 1:** XPS elemental speciation percentage within fungal melanins and p-1,8-DHN

	Carbon			Nitrogen	Oxygen	
	C–C/ C = C	C–O/ C–N	C = O/ C = N	–NCR_2_ (R = H,C)/ Amide	O = C/ Oxide	O–C/ O–H
Fungal melanin – 1 st extraction	63%	15%	4%	3%	12%	3%
Fungal melanin – 2nd extraction	48%	17%	10%	2%	9%	14%
p-1,8-DHN	45%	27%	5%	0%	3%	20%

**Fig. 4:**
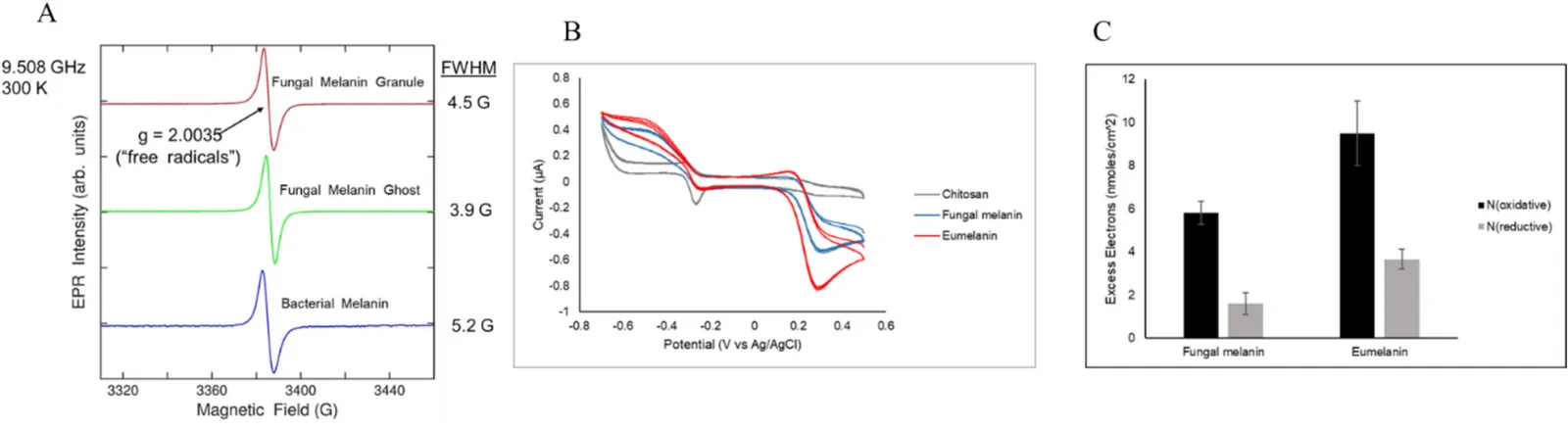
Characterization of redox properties of *E. viscosa* melanin. (**A**) EPR characterization of fungal melanin granule, fungal melanin ghost, and bacterial melanin (eumelanin). (**B**, **C**) Cyclic voltammetry analysis of fungal melanin and eumelanin. A chitosan film without melanin was used as a null control. Three replicate samples of each type of melanin were measured. The *p*-values for the comparison of the N (oxidation) values and N (reductive) values between fungal melanin and eumelanin by a two-tailed *T*-test are 0.0009 and 0.0844, respectively

Stable free radicals are characteristic of melanin and have a unique electron paramagnetic resonance (EPR) signal. To examine this signal in fungal melanin, equal amounts of extracted melanin granules, fungal melanin ghosts, and eumelanin synthesized from the recombinant bacterium (Wang et al. 2020) were examined using EPR (Fig. 4A). These samples exhibited distinct, stable free radical signals with Zeeman splitting g-values of 2.0035.

We analyzed the redox properties of melanin using the semi-quantitative redox probing method. When compared with synthetic eumelanin derived from bacterial sources (Wang et al. 2020), both produced comparable cyclic voltammograms when probed with ferrocene and phenazine-1-carboxylic acid (Fig. 4B). However, when quantified for excess electron exchanges, fungal melanin exhibited a significantly lower oxidative capacity while maintaining a comparable reducing capacity (Fig. 4C). This observation indicates that fungal melanin possesses a lower overall redox activity than eumelanin. The decreased redox activity of fungal melanin is likely attributable to the presence of chitin within its structure.

### Genetic verification of the DHN-melanin biosynthesis pathway

**Fig. 5:**
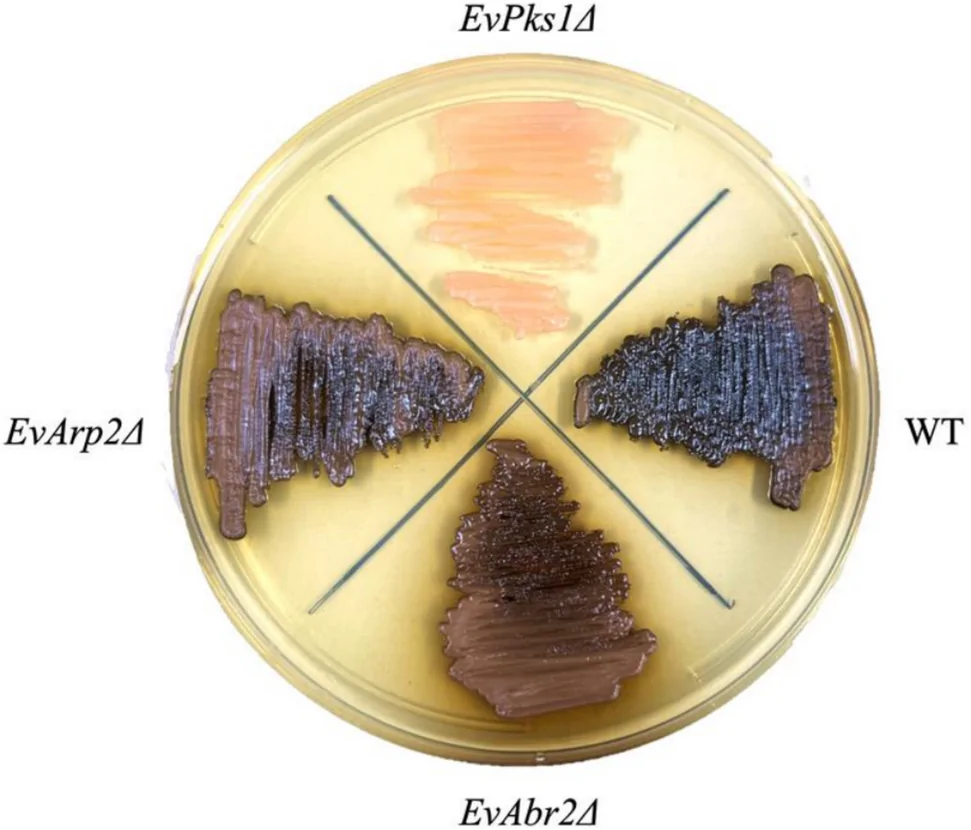
Colony phenotypes of *E. viscosa* wild-type and three genetic mutants (*EvPks1Δ*, *EvArp2Δ*, and *EvAbr2Δ*) in the DHN-melanin biosynthesis pathway

The DHN/allomelanin biosynthesis pathway was identified from the genome of *E. viscosa* (Carr et al. 2023). To characterize the impact of melanin on the phenotypic properties of *E. viscosa*, a melanin-deficient mutant was generated by deleting *EvPks1*, a gene encoding the polyketide synthase involved in the initial step of melanin synthesis. The gene deletion cassette, created using fusion PCR, was transformed into competent *E. viscosa* cells via electroporation, as described in our previous study on the black yeast *E. lecanii-corni* (Romsdahl et al. 2021). Although only 12 transformants were obtained from each transformation, 8 of these were melanin-deficient colonies, indicating a low transformation efficiency but a high efficiency (66.7%) of site-specific integration of the cassette into the genome. One of the melanin-deficient colonies was streaked onto a YPD agar plate, displaying a pink color (Fig. 5) similar to the melanin-defective mutant generated through mutagenesis with the alkylating agent ethyl methanesulfonate (EMS) (Carr et al. 2023). This observation suggests that the pink carotenoid pigment is highly produced by *E. viscosa* but is masked by abundant melanin in the wild-type strain.

Additionally, two other genes in the DHN-melanin biosynthesis pathway, *Arp2* (encoding scytalone dehydratase) and *Abr2* (encoding laccase), were deleted. The resulting mutants were confirmed with the site-specific PCR (Fig. S1). Both deletions showed similar efficiencies for transformation and site-specific integration as observed in the *EvPks1Δ* deletion. The resulting mutants (*EvAbr2Δ* and *EvArp2Δ*) exhibited dark brown pigmentation, corresponding to the accumulation of intermediate compounds 1,3,8-trihydroxynaphthalene (1,3,8-THN) and 1,8-dihydroxynaphthalene (1,8-DHN) (Fig. 5).

### Phenotypic analyses of *E. viscosa* and its melanin deficient mutant Evpks1

**Fig. 6:**
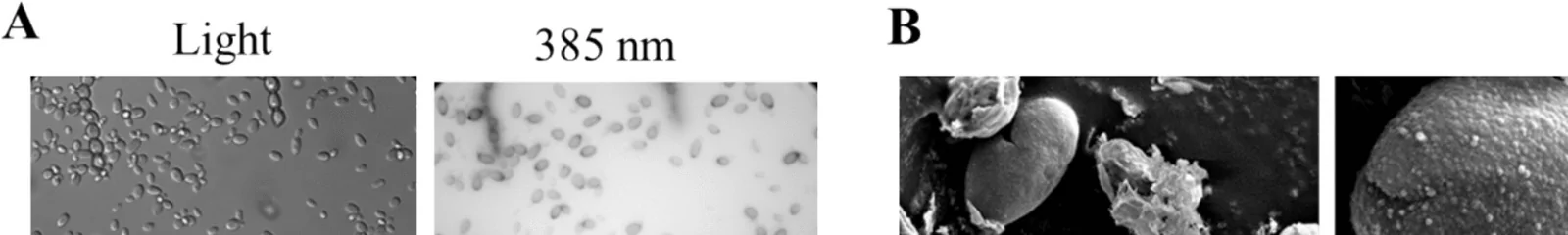
Cellular phenotypes of *E. viscosa* wild-type and *Pks1Δ* mutant. (**A**) Microscope images at visible Light and 385 nm wavelength; (**B**) FESEM images

Like many other black yeasts, *E. viscosa* is dimorphic, exhibiting both yeast and pseudohyphae forms. However, the yeast form appears dominant in both the wild-type and *EvPks1Δ* mutant when grown in YPD liquid media (Fig. 6A). Interestingly, under the microscope using a 385 nm wavelength, which is optimally absorbed by melanin, *EvPks1Δ* cells were invisible, whereas wild-type cells were clearly observed with black melanin outlined on their cell walls (Fig. 6B). It was also noted that the density of melanin was substantially higher at the budding sites of yeast cells, suggesting that melanin synthesis might be actively occurring during cell division. Furthermore, both types of cells were analyzed by field emission scanning electron microscopy (FESEM). SEM images revealed typical yeast morphologies with defined nano-sized melanin granules on the surface of wild-type cells (Fig. 6B). In contrast, the surface of the *EvPks1Δ* mutant cells appeared very smooth (Fig. 6B). These microscopic images confirmed that melanin was completely abolished from the cell wall in the *EvPks1Δ* mutant.

### Effect of melanin on stress resistance in *E. viscosa*

**Fig. 7:**
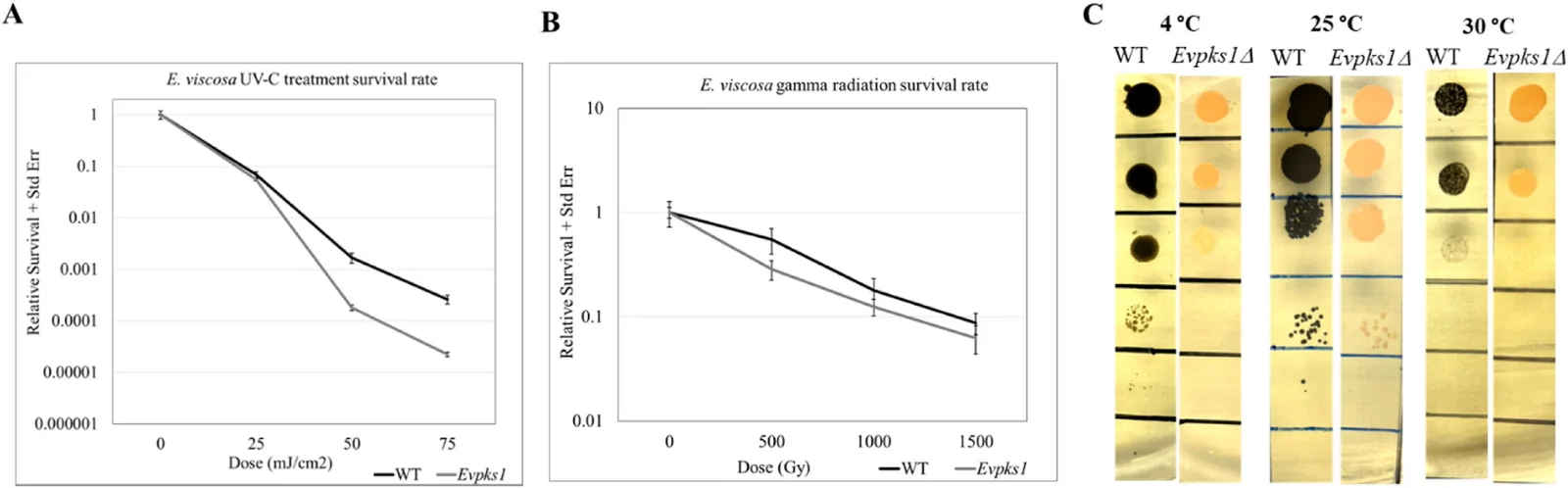
Survival of *E. viscosa* WT and *Pks1Δ* cells following exposure to varying total doses of (**A**) UV-C radiation with a dose rate of 0.273 mJ/cm^2^ s^−1^, (**B**) γ-radiation with a dose rate of 22.7 Gy/min, and (**C**) varying temperatures (the representative of three biological replicates)

In our previous studies, melanin in two other *Exophiala* species was shown to confer protection against UV and ionizing radiation (Romsdahl et al. 2021; Schultzhaus et al. 2020). Similarly, the wild-type *E. viscosa* strain and the melanin-deficient *Pks1Δ* mutant were exposed to varying doses of UV-C (5–75 mJ/cm^2^) and γ-radiation (500–1500 Gy), respectively. As expected, the *EvPks1Δ* mutant was more susceptible to UV-C than the wild-type (Fig. 7A), though the reduction in viability was less pronounced compared to that observed in *E. lecanii-corni* counterparts (Romsdahl et al. 2021). When exposed to γ-radiation, *E. viscosa* demonstrated a D10 value (the dose at which only 10% of cells survive) of approximately 1500 Gy, which is higher than that of *E. lecanii-corni* (D10 ~ 1000 Gy) (Romsdahl et al. 2021) but considerably lower than *E. dermatitidis* (D10 ~ 3000 Gy) (Schultzhaus et al. 2020). Interestingly, in contrast to *E. lecanii-corni*, the absence of melanin in *E. viscosa* resulted in only a moderate reduction in resistance to γ-radiation across doses of 500, 1000, and 1500 Gy (Fig. 7B). These findings suggest that melanin may not play a dominant role in protecting *E. viscosa* cells against high doses of ionizing radiation. Instead, other underlying mechanisms, such as the production of carotenoids, might contribute significantly to radiation protection in this species.

A previous study showed that *E. viscosa* is capable of growing at temperatures ranging from 4 °C to 27 °C (Carr et al. 2023). To investigate the role of melanin in growth, equal numbers of cells from the wild-type strain and the *EvPks1Δ* mutant were diluted, spotted, and incubated on YPD agar plates in the dark at 4 °C, 25 °C, and 30 °C (Fig. 6C). Both strains grew equally well at 25 °C, but their growth was noticeably reduced at 30 °C, suggesting susceptibility to elevated temperatures. Interestingly, at 4 °C, the wild-type strain exhibited approximately tenfold higher growth compared to the melanin-deficient mutant. This suggests that melanin may help retain or capture heat in cold environments, supporting fungal survival. These findings are consistent with the previous report that melanin can impact fitness of fungal cells (Cordero et al. 2018).

## Discussion

The findings of this study provide significant insights into the biosynthesis, chemical properties, and functional roles of DHN-melanin (allomelanin) in *Exophiala viscosa*. Using a YPD medium, we successfully extracted melanin from fungal cultures, achieving a yield of approximately 8 g/L including both melanin granules and melanin ghosts. This highlights the potential of *E. viscosa* as a robust model organism for allomelanin production. Currently, the majority of extracted melanin consists of melanin ghosts, indicating that a large portion remains trapped in the fungal cell wall due to strong interactions with structural components such as chitin and glucan, as evidenced by the formation of melanin ghosts (Camacho et al. 2019; Wang et al. 1996) (Fig. 2B). Breaking the cell wall through enzymatic or mechanical methods could release melanin, thereby enhancing the overall yield of extracted melanin. Additionally, extracellular vesicles (EVs) have been implicated in fungal melanization and melanin excretion (Eisenman et al. 2009; Upadhyay et al. 2016; Walker et al. 2010). Previous studies demonstrated that increasing peptone or tyrosine levels in the growth medium induced melanin excretion from *E. viscosa* (Carr et al. 2023), suggesting that EVs may serve as a delivery system to transport melanin outside fungal cells. Understanding the EV-mediated excretion pathway in this fungus is a promising strategy to further enhance melanin production. By optimizing growth conditions and identifying inducers of melanin excretion, it may be possible to increase the efficiency of EV-based melanin release, leading to higher production yields of melanin granules.

The FTIR and XPS analyses highlight similarities between *E. viscosa* melanin and synthetic allomelanin, such as the presence of hydroxyl and aromatic groups, while also identifying chitin residues as a unique component. The association with chitin was further validated by ssNMR, which confirmed the integration of aliphatic and aromatic carbon environments consistent with DHN-melanin. Similar findings have been reported in other melanized fungi (Camacho et al. 2019; Singla et al. 2021), suggesting that melanin either incorporates cell wall components during excretion or detaches from the melanized cell wall during cultivation. This chitin association likely introduces impurities that can affect melanin’s solubility, stability, and functional properties such as lower oxidative capacity (Fig. 4B), particularly its role in fungal cell wall integrity and environmental resilience. To enhance the purity of allomelanin, incorporating cell wall-degrading enzymes, such as chitinase, or extending acid–base cycling steps during the extraction process may prove effective. Alternatively, the incorporation of melanin into the fungal cell wall imparts a rigid structure, leading to the formation of melanin ghosts, which retain the cellular morphology even after harsh acid/base digestion of melanized cells. The unique spatial structure of melanin ghosts has demonstrated advantages in various applications, including radiation shielding, radical scavenging, and chemical adsorption (Dadachova et al. 2008; Kim et al. 2017; McCallum et al. 2021). In this context, the production of melanin ghosts from black yeast cultures presents an additional avenue for melanin biomanufacturing.

The deletion of key genes in the DHN-melanin biosynthesis pathway (*EvPks1Δ*, *EvArp2Δ*, and *EvAbr2Δ*) confirm the genetic basis of melanin production in *E. viscosa*. Phenotypic analysis of the *EvPks1Δ* mutant demonstrate melanin's essential role in maintaining cell wall structure and integrity, as evidenced by the mutant strain's smooth cell surface and the absence of melanin. These findings are consistent with previous studies highlighting the structural importance of melanin in black yeasts. Unlike melanized filamentous fungi, which synthesize DHN-melanin differentially in conidia, black yeast *Exophiala* species produce melanin constitutively throughout their development. In other fungi, such as *Aspergillus* and *Penicillium* species, gene deletions in the DHN-melanin biosynthesis pathway lead to distinct pigmentation changes, ranging from yellow to brown, due to the accumulation of intermediate molecules with varying light absorption properties (Cleere et al. 2024; Jackson et al. 2009; Tsai et al. 1999). In our study, *EvArp2Δ* and *EvAbr2Δ* mutants initially exhibited a light brown color, which darkened to dark brown after seven days of incubation. This suggests that in the absence of functional Arp2 or Abr2, intermediates such as 1,3,6,8-T4HN and 1,8-DHN may undergo spontaneous oxidation and polymerization, forming a melanin-like pigment. Additionally, other enzymes in *E. viscosa* may partially compensate for the loss of Arp2 or Abr2, facilitating the accumulation and eventual polymerization of downstream intermediates into dark brown pigment. Successfully deleting these three genes with comparable transformation and homologous recombination efficiencies not only validated the DHN-melanin biosynthesis pathway but also highlighted the potential of genetically engineering *E. viscosa* for diverse applications.

Our results confirm that melanin provides UV protection in *E. viscosa*, consistent with observations in other *Exophiala* species. However, the moderate reduction in γ-radiation resistance in the *EvPks1Δ* mutant suggests that melanin may not be the sole determinant of radiation protection in this species. The production of carotenoids, as indicated by the pink pigmentation in melanin-deficient mutants, likely plays a complementary role, highlighting the multifaceted stress tolerance mechanisms in *E. viscosa*.

Our study reveals that wild-type *E. viscosa* exhibited a growth advantage over the melanin-deficient mutant *EvPks1Δ* at 4 °C in the dark, but not at 30 °C. Cordero et al. previously reported that melanin-mediated heat capture from visible light enhanced the fitness of melanized yeast at low temperatures, but had negative effects at higher temperatures (Cordero et al. 2018), suggesting that melanin can absorb and convert solar energy into heat. However, in our study, the growth assays were conducted entirely in the dark, with agar plates wrapped in aluminum foil to exclude any interaction with electromagnetic radiation. This indicates that the observed growth advantage of black yeast is independent of light-driven energy capture. Instead, our findings suggest that melanin may possess broad absorption properties and a high heat capacity, allowing it to trap and retain ambient thermal energy from the environment. This retained heat may help stabilize the local microenvironment, improving membrane fluidity and metabolic activity at low temperatures. Additionally, the known free radical scavenging property of melanin may help mitigate oxidative stress associated with cold exposure (Chattopadhyay et al. 2011). Taken together, these results highlight the potential of melanin as a functional component in the development of materials for thermal regulation.

The robust growth of *E. viscosa* under laboratory conditions, its ability to produce melanin, and its non-pathogenic nature position it as an attractive candidate for industrial applications. The genetic tractability demonstrated in this study paves the way for further metabolic engineering to enhance melanin production or tailor its properties for specific applications. For instance, introducing strong fungal promoters to drive high expression of key genes in the DHN melanin biosynthesis pathway, along with deletion of pathway repressors, could enhance melanin yield. Additionally, integration of a gene encoding a glycosyltransferase may enable the production of a melanin derivative with improved solubility.

While this study establishes *E. viscosa* as a promising model for melanin research, several challenges remain. The observed chitin association highlights the necessity for further purification methods to obtain melanin with higher purity for specific applications. Additionally, the structural and functional role of melanin in conjunction with other stress tolerance factors, such as carotenoids, warrants deeper investigation.

In summary, this work not only advances our understanding of fungal melanin and demonstrates the potential of *Exophiala viscosa* as a versatile system for producing and studying allomelanin, but also underscores the potential for translational applications beyond fundamental fungal biology. The unique physicochemical properties of fungal allomelanin, including its microporosity, redox activity, and radiation-absorbing capacity, highlight its potential for diverse applications in materials science. Melanin ghosts, with their preserved structural integrity and stability under extreme conditions, offer promising opportunities for developing bio-derived materials for radiation shielding, environmental remediation, and advanced coatings. Moreover, the scalable production of allomelanin in a genetically tractable and non-pathogenic host like *E. viscosa* paves the way for sustainable biomanufacturing platforms capable of producing functional biomaterials tailored for space exploration, biomedicine, and ecological engineering.

## Supplementary Information

Below is the link to the electronic supplementary material.

Supplementary file1 (DOCX 2.12 MB)

## Data Availability

No datasets were generated or analysed during the current study.
