# Supplementary material for: Genetic, structural, and functional characterization of allomelanin from black yeast Exophiala viscosa, a chassis for fungal melanin production
Source: Appl Microbiol Biotechnol. 2025 Oct 8;109(1):216. doi: 10.1007/s00253-025-13597-w (PMC12507995; doi:10.1007/s00253-025-13597-w)
Supplement: Supplementary file 1 — Supplementary file1 (DOCX 2.12 MB) [file 253_2025_13597_MOESM1_ESM.docx]

**Supplemental Materials**

**Table S1 Primers for gene deletions**


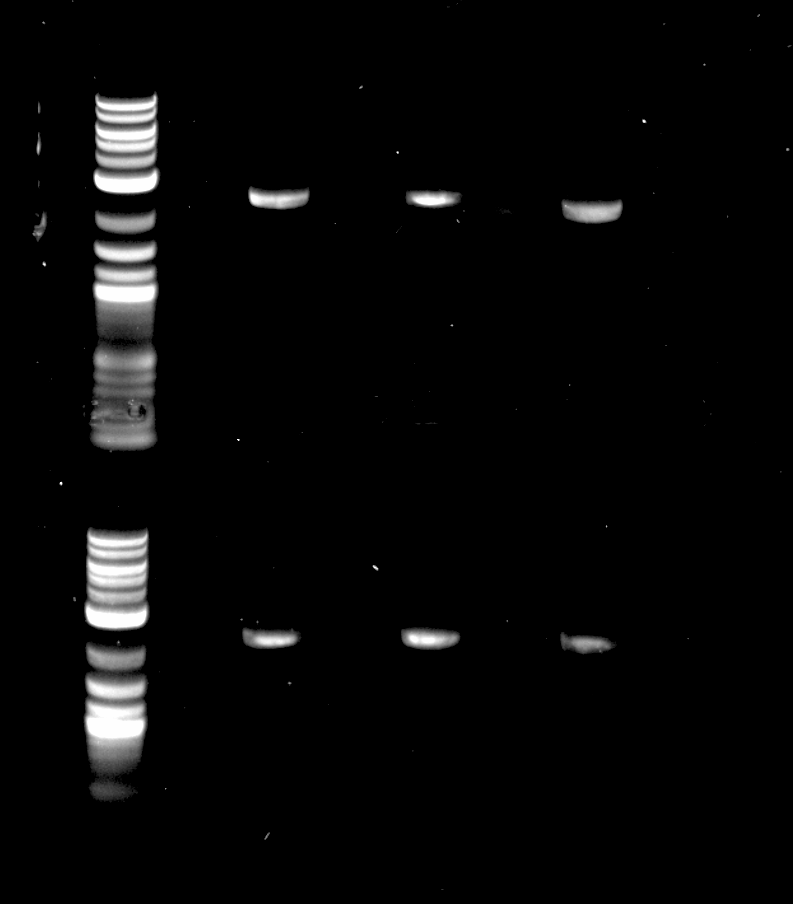


1 2 3 4 5 6

**Figure S1**. PCR confirmation of gene deletion mutants *Evpks1Δ*, *Evabr2Δ and Evarp2Δ.* Top gel: Lane 1: wild type, pks1_F2/hph_R; Lane 2: *Evpks1*, pks1_F2/hph_R; Lane 3: wild type, arp2_F2/hph_R; Lane 4: *Evarp2*, arp2_F2/hph_R; Lane 5, wild type, abr2_F2/hph_R4, Lane 6: *Evabr2*, abr2_F2/hph_R.

Bottom gel: Lane 1: wild type, hph_F/pks1_R5, *Evpks1*, hph_F/pks1_R5; Lane 3: wild type, hph_F/arp2_R5; Lane 4: *Evarp2*, hph_F/arp2_R5; Lane 5, wild type, hph_F/abr2_R5, Lane 6: *Evabr2*, hph_F/abr2_R5.

Bands consistent with amplicon sizes confirmed replacement of the targeted gene with the selectable marker, hph. Wild type was not amplified by any pair of primers.
